# Cold stress during winter over North India: Patterns, trends, and mortality risks

**DOI:** 10.1371/journal.pone.0351740

**Published:** 2026-06-22

**Authors:** K. S. Athira, Raju Attada, Sulekha Komeravelli

**Affiliations:** Department of Earth and Environmental Sciences, Indian Institute of Science Education and Research Mohali, Mohali, Punjab, India; Yunnan University, CHINA

## Abstract

The cold temperatures during winter contribute to significant discomfort, causing cold stress to the lives of people. In this study, we investigate the cold stress conditions and their trends over north India during November – February for the period 1982−2020, using Universal Thermal Climate Index (UTCI), a widely recognized biometeorological variable. The onset of cold stress typically initiates in the late evening (18 IST) and gradually spreads across the north India. It then intensifies over night into moderate cold stress, persisting from 20 IST until the early morning hours (7 IST). Cold stress is found to be most intense in January, with UTCI values ranging between 0 °C to −13 °C among winter months. During cold wave events, cold stress reaches its maximum spatial extent and intensity throughout the study region, with the most intense conditions observed in Jammu & Kashmir and Ladakh. Results show a significant increase in slight cold stress hours during the winter season over the period 1982−2020 in north India. This increase reflects a transition from stronger cold stress categories to the slight cold stress category. Such a shift indicates a reduction in the severity of cold stress rather than an intensification of cold conditions. Slight cold stress occurs when UTCI values range between 0 °C and −9 °C and the duration of such conditions has increased at a rate of 33.64 hours per decade. This increase is most prominent in the high-altitude regions of Jammu & Kashmir, Ladakh and Himachal Pradesh. In contrast, moderate cold stress which occurs when the UTCI values are in the range −9 °C to −13 °C shows a significant decreasing trend, particularly in Rajasthan, Punjab, Haryana and Uttar Pradesh. Despite this, cold stress remains a major contributor to cold wave-related mortality in north India. Uttar Pradesh recorded the highest number of deaths of 4449, followed by Punjab (2606) and Bihar (2479). Although these states generally experience only slight cold stress with UTCI values in the range of 0 °C to 9 °C, the impact is amplified by wind chill with a category of ‘Tonic- very cold sub comfort’ caused by cold, dry northwesterly winds blowing directly into the region. This compound effect of cold stress and wind chill considerably enhances the mortality risk during cold wave events across north India.

## 1. Introduction

During the winter months, north India experiences significant drops in temperature, often resulting in cold wave events accompanied by cold northerly winds. These cold waves are typically triggered by the passage of western disturbances and tend to intensify after the disturbance exits the region [1]. Large-scale atmospheric phenomena, such as atmospheric blocking, further prolong and amplify the severity of these events, affecting broader areas of north India [2]. Despite a decreasing trend in the frequency of cold waves in recent decades [3,4], the occurrence of extreme cold wave events remains a serious concern [5,6]. The persistence of cold temperatures during such events significantly disrupts daily life and poses a severe health risk, particularly for vulnerable groups such as the elderly, children [7] and individuals lacking adequate shelter and clothing. In extreme cases, cold waves can be fatal.

Cold waves have a major impact on people in India. North India accounts for nearly half of all cold wave-related deaths in the country, followed by eastern states [8]. Between 1970 and 2019, Bihar, Uttar Pradesh (UP), Jharkhand, West Bengal, and Rajasthan recorded the highest numbers of cold wave-related fatalities [9]. The study by Ray et al. [10] pointed out that the effects due to cold exposure are often detrimental in lower-latitude countries with milder winter climates, where populations are less adapted to cold conditions. This may also be the case in India, where lower physiological adaptation and fewer protective measures are available in such a climate.

Cold exposure is linked to cardiovascular and respiratory mortality [11,12], with effects that often linger several days or weeks after a cold wave event [13–15]. This delayed and long-lasting physiological stress increases vulnerability among people with pre-existing health conditions [16–18]. According to Lu et al. [19], India ranks second globally in terms of the expected annual population affected by cold waves. From 1978 to 2014, six hundred six (606) cold wave events caused approximately 8,520 deaths, with Bihar and UP alone accounting for 75% of the fatalities [20].

A recent study in India further highlighted the gender and age disparities in vulnerability to cold stress. For instance, [21] found that males experienced four to seven times higher cold-related mortality than females. Cold-related deaths were most common among adults aged 45–60, followed by the elderly and those aged 30–45. Similarly, a study in Pune reported that during the period 2004–2012, cold accounted for approximately 6% of all deaths, almost five times higher than heat-related deaths [22]. In Delhi, the likelihood of experiencing slight cold stress during cold nights was found to be nearly 59% higher than in other metropolitan cities like Mumbai, Kolkata, and Chennai [23].

To properly assess the effects of cold waves on health, it is imperative to use robust thermal stress indices. The Universal Thermal Climate Index (UTCI) is a comprehensive biometeorological index based on human thermal balance. Unlike basic air temperature measures, UTCI accounts for meteorological parameters as well as human physiological responses, making it a valuable tool to assess human thermal comfort under cold stress conditions [24,25]. For example, a study in Poland found significantly increased hospital admissions for hypertension during cold stress, with women more affected than men [25]. Similarly, Sheffield and Landrigan [26] highlighted that children who spend more time outdoors during winter are also highly susceptible to cold stress. Moderately cold temperatures (13.8 °C to minimum mortality thresholds) are associated with a higher mortality burden than extreme cold (<13.8 °C) or extreme heat, especially for causes such as stroke and respiratory illness. In 2015, an estimated 197,000 deaths from stroke, ischaemic heart disease, and respiratory diseases in India among adults aged 30–69 were attributed to moderately cold temperatures [27]

Despite these alarming figures, research on cold stress and mortality associated with cold waves in India remains limited. While few studies have assessed thermal stress using the UTCI in India [28–30], dedicated investigations into cold stress patterns, their temporal and spatial trends, and their links to mortality are still lacking. This gap is particularly significant because cold stress plays a central role in exacerbating health risks during cold waves. Understanding cold stress is therefore essential for quantifying the burden of cold wave-related mortality in north India, where severe winter extremes pose serious risks to vulnerable populations. To address this gap, the present study analyzes patterns and variability of cold stress conditions across India using the UTCI during the winter season (November-February) over the period 1982–2020. It further examines cold wave-related mortality in north Indian states to better understand the public health implications of cold stress.

The paper is organized as follows. Section 2 describes the data and methods employed in the study. Section 3 presents the results, which are detailed in three subsections. Specifically, Section 3.1 examines the spatial patterns and variability of cold stress conditions across north India, Section 3.2 analyzes the trends in cold stress during the study period and Section 3.3 investigates the mortality risks associated with cold waves. Finally, Section 4 provides a summary of the main findings.

## 2. Data and methods

### 2.1. Data used

Minimum temperature is obtained from IMD available at 1° × 1° resolution for the period 1982–2020. Horizontal wind components are utilized from the fifth-generation European Centre for Medium-Range Weather Forecasts (ECMWF) atmospheric reanalysis (ERA5); [31] available at 0.25° × 0.25° resolution at every hour from 1982–2020. The UTCI is obtained from the ERA5-HEAT dataset at 0.25° × 0.25° resolution at every hour available at Copernicus Climate Change Service [32]. UTCI classifies environmental conditions into 10 thermal stress categories, ranging from “no thermal stress” to “extreme cold” or “extreme heat”, providing a standardized measure for evaluating thermal comfort. Mortality data of cold waves from 2000 to 2020 is obtained from Indiastat.com (https://www.indiastat.com/data/meteorological-data/heat-cold-waves). Long-term cold wave mortality data (1980–2020) are obtained from IMD disastrous weather events (https://imdpune.gov.in/library/public/publication.html).

### 2.2. Methodology

The diurnal variability of UTCI is studied by computing the long-term mean of hourly UTCI. The UTCI is defined as the air temperature (Ta) under reference conditions that produces the same physiological response as the current actual conditions [33,34]. According to the UTCI classification, thermal conditions between +9 °C and +26 °C are considered comfortable, with no thermal stress. When temperatures fall between +9 °C and 0 °C, individuals may experience slight cold stress, while values ranging from 0 °C to –13 °C correspond to moderate cold stress. Strong cold stress occurs within the range of –13 °C to –27 °C, and very strong cold stress is observed between –27 °C and –40 °C. Any temperature below –40 °C represents extreme cold stress. We further computed the WCI, as suggested by [35,36], by the following equation:


$$\begin{array}{c} K=\text{1}.\text{1}\text{6}\;\times [\left(\text{1}\text{0}.\text{4}\text{5}\;+\text{1}\text{0}U^{\frac{\text{1}}{\text{2}}}\;-U)\right)(\text{3}\text{3}\;-Ta)] \end{array}$$ (1)

Where K is WCI in watts/m^2^, U is wind speed in m/s, and Ta is air temperature (°C).

The wind chill temperature in Watt/m^2^ represents the rate of heat transfer from the body to the environment [37]. A factor of 1.16 is multiplied to convert into SI units of watts/m^2^ [37]

Siple and Passel’s [35] cooling experiment calculated heat transfer coefficients, referred to as wind chill factor, $h_{wc}$. They related $h_{wc}$ to the wind speed by


$$\begin{array}{c} h_{wc}\;=\text{1}\text{0}.\text{4}\text{5}+\text{1}\text{0}U^{\frac{\text{1}}{\text{2}}}-U \end{array}$$

The wind chill index (K), represents an estimation of heat transfer from the human body, was calculated as the product of the wind chill factor ($h_{wc}$) and the temperature difference between an assumed skin temperature of 33 °C and the ambient air temperature (Ta) [37]. The Siple-Passel wind chill index effectively captures the wind chill effect over North India, as evident from the diurnal variability of WCI. During cold wave conditions, WCI values typically range between 600–800 Wm ⁻ ² and 800–1000 Wm ⁻ ², corresponding to cold comfort and very cold sub-comfort categories, respectively. These conditions are primarily associated with strong northwesterly winds, with wind speeds exceeding 4 ms ⁻ ¹. However, the study by Chowdhury and Gore [38] observed that the applicability of the wind chill index is limited by its strict criteria; air temperature must be lower than the assumed skin temperature of 33 °C and wind speed must exceed 1.34 ms ⁻ ¹. As a result, only a small number of days, predominantly night-time periods, qualify as wind chill days in the study region.

The wind chill index is classified into six categories: Class 0 (Endothermal) – very hot discomfort (K ≤ 200); Class 1 (Atonic) – hot discomfort (200 < K ≤ 400); Class 2 (Hypotonic) – hot sub-comfort (400 < K ≤ 600); Class 3 (Cold) – comfort (600 < K ≤ 800); Class 4 (Tonic) – very cold sub-comfort (800 < K ≤ 1000); and Class 5 – very cold discomfort (K > 1000). It is to be noted that wind chill, also known as wind’s chill-producing effect, refers to the combined influence of wind and air temperature on the rate of heat loss from the human body and other organisms. It is used to represent how these factors together reduce thermal comfort. Importantly, wind chill is not the actual temperature, but rather an index that reflects the perceived temperature as experienced by the body under the combined effect of wind and temperature [39]. Lastly, the trends of the cold stress are analysed using the Mann-Kendall test.

## 3. Results and discussion

### 3.1. Patterns and variability

**Fig 1:**
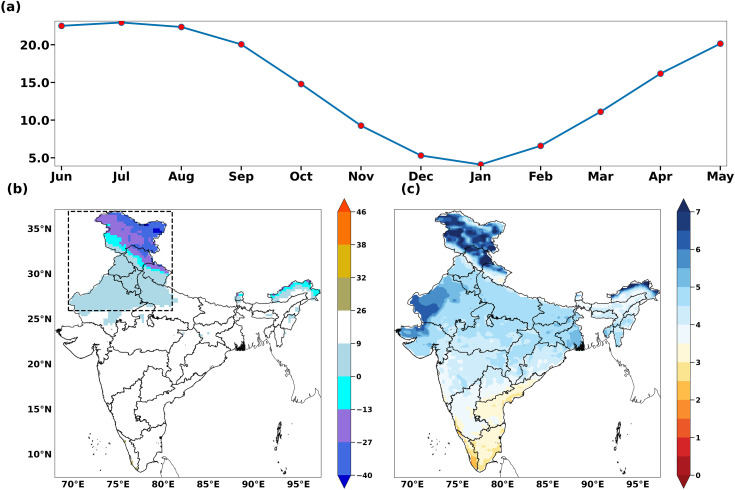
Distribution of Minimum Temperature (°C) and UTCI (°C). (a) Annual cycle of minimum temperature (°C) over north India (69.5°E-81°E, 26°N-37°N) during November-February for the period 1982–2020, (b) climatology of UTCI (°C) and (c) standard deviation of UTCI. Administrative boundary data were obtained from GeoBoundaries (https://www.geoboundaries.org/) under the Creative Commons Attribution 4.0 International (CC BY 4.0) license. The map was generated by the authors.

The minimum temperature in north India exhibits a considerable reduction during the winter months from November to February. This is evident in the annual cycle of minimum temperature (Fig 1a), which shows a dip as it approaches winter, starting in November and lasting until February, when the temperature drops from 12 °C to a minimum of 7 °C during the coldest month of January.

The northern parts of the country experience winter surface temperatures largely between 15 and 24 °C, with considerably lower temperatures in the hilly region [40]. Climatologically, north India’s minimum temperature decreases during winter from parts of Madhya Pradesh to the extreme northern states, such as Himachal Pradesh (HP) and Jammu and Kashmir (J&K) [2]. It is noticeable that the lowest minimum winter temperature in India is observed in the northern part of Kashmir, which ranges from 275 to 285 K. All these conditions provide favourable conditions for the development of cold stress conditions in north India [41].

The climatology of UTCI (Fig 1b) indicates that the states of Rajasthan, Punjab, Haryana, UP, Uttarakhand, HP and J&K are the major cold stress hotspots of the country, with conditions varying from slight to extreme. Populations in these regions are particularly vulnerable, as cold stress represents a sustained physiological strain on the human body arising from the combined effects of environmental factors such as air and radiant temperature, wind speed and humidity, along with personal factors such as clothing insulation and activity level. Together, these factors can cause a decline in body temperature. The impacts of cold stress can lead to discomfort, reduced attention, diminished ability to carry out tasks, deterioration of health, cold-related injuries, and even death [42]. The rest of the country generally does not experience cold stress conditions during winter, but very few regions in Kerala witness moderate heat stress during this time. The standard deviation of UTCI is maximum in the regions of Rajasthan, J&K, parts of HP and Uttarakhand (Fig 1c). This signifies that the people in these regions experience variability in thermal stress during winter. Based on these observations, we selected a region (shown as a dashed black box in Fig 1c) to study the variability and trend of winter cold stress in north India. Studying the effects of cold in this region is crucial, as it is the most densely populated part of the country, leaving more people exposed to cold stress conditions, which exerts severe consequences on people’s health.

**Fig 2:**
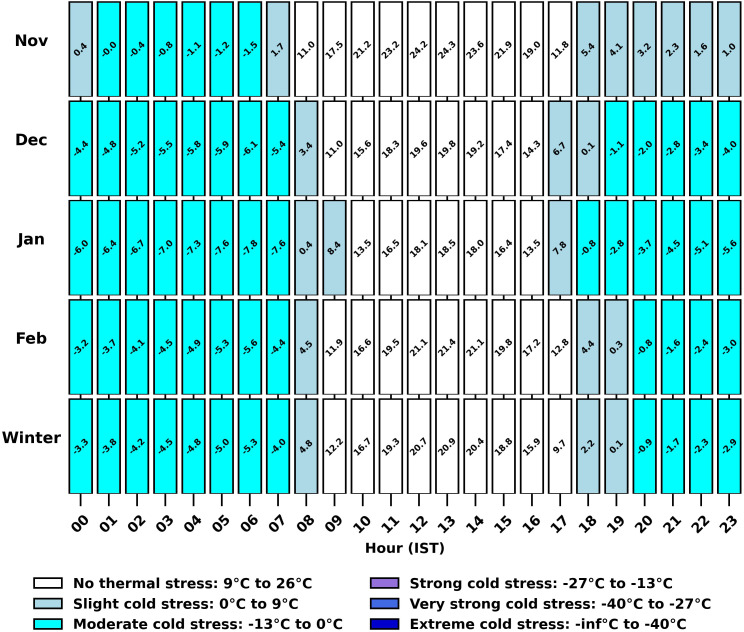
Diurnal variability of UTCI (°C) during winter months (November, December, January and February) and the winter season over north India.

For this purpose, we analysed the diurnal and seasonal variation of UTCI in north India for the winter months of November to February, as shown in Fig 2.

During winter, cold stress is most pronounced from late evening (18 IST) to early morning (7 IST), with moderate cold stress occurring between 20 IST to 7 IST. A similar night-time dominance of cold stress has been reported for Central Europe, where extreme cold stress occurs between 6 p.m. and 6 a.m., associated with the inflow of frosty Arctic and polar continental air masses [43].

After 8 IST, UTCI values rise, leading to a reduction in cold stress, transitioning to no thermal stress (9 °C to 26 °C). Among the winter months, January experiences the most intense cold stress, with most hours of the day experiencing moderate cold stress conditions (−13 °C to 0 °C), followed by December and February. Pappenberger [44] noted that in January, vast regions of Asia and North America experience persistent strong cold stress. On the contrary, November shows only slight cold stress during most times of the day. This pattern highlights the diurnal and monthly variation of cold stress in north India during winter, with the most intense cold stress occurring during night-time and early morning hours during the peak winter months of January and December.

We further analysed the spatial diurnal variability of cold stress for individual winter months to identify the specific regions of north India affected by varying intensities of cold stress at different times of the day. In November (S1 Fig), cold stress is not felt in north India, except in parts of J&K, Ladakh and HP, where moderate to strong cold stress exists only during the late evening and night hours and slight cold stress during the rest of the day. In the month of December (S2 Fig), slight cold stress begins in north India at 19 IST and gradually spreads across the entire north Indian region, which abates after 8 IST in the morning. In J&K and HP, cold stress persists throughout the day, with strong-very strong cold stress occurring between 18 IST and 7 IST.

**Fig 3:**
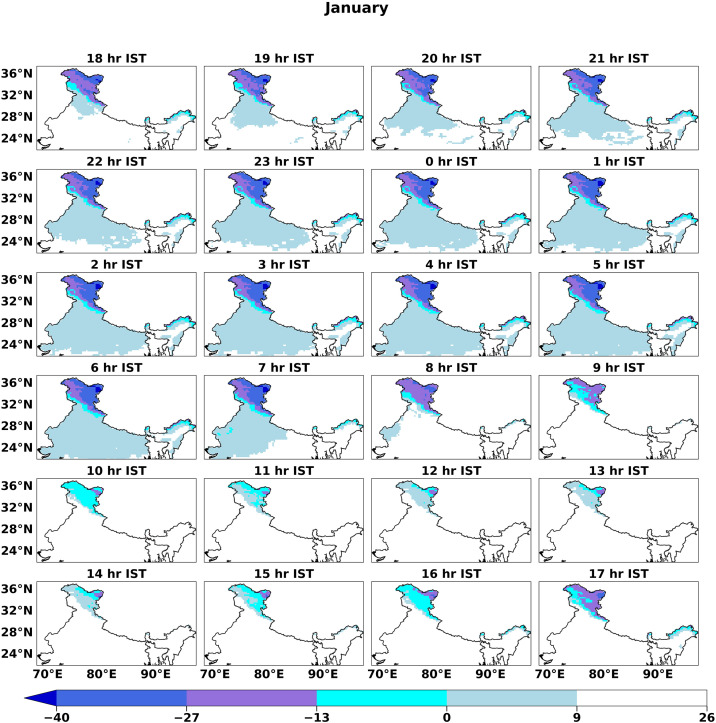
Diurnal variability of cold stress during peak winter month, i.e., January (°C). Administrative boundary data were obtained from GeoBoundaries (https://www.geoboundaries.org/) under the Creative Commons Attribution 4.0 International (CC BY 4.0) license. The map was generated by the authors.

With the drop in temperatures, most parts of north India experience cold stress in January (Fig 3).

During the evening and night (18 IST – 6 IST), cold stress intensifies, with strong to extreme cold stress observed in J&K, Ladakh, HP and Uttarakhand. Other northern states like Rajasthan and UP experience slight cold stress, with some isolated pockets of Rajasthan witnessing moderate cold stress in the early morning hours. After 8 IST, UTCI values gradually increase, causing cold stress to subside, though high-altitude regions continue experiencing cold stress. The most intense conditions occur between midnight and early morning. J&K, Ladakh and HP remain under moderate cold stress throughout the day in January.

In February (S3 Fig), slight cold stress begins around 21 IST – 22 IST, initially affecting J&K, HP and parts of northern Rajasthan and UP. By 5 IST, it extends across most of north India, with some regions experiencing moderate cold stress. As temperatures rise after 7 IST, cold stress conditions subside. J&K and HP consistently experience moderate cold stress throughout the day, particularly from evening 18 IST to early morning 6 IST. In contrast, other parts of north India face slight cold stress during night-time and early morning, which dissipates after sunrise.

As the temperatures rise after January, cold stress in February is less intense and shorter in duration. Moderate to very strong cold stress conditions continue to prevail over the high altitude regions of J&K and HP during late evening to early morning. For the rest of north India, slight cold stress only initiates around 20 IST and gradually spreads without covering the whole of north India. Hence, from the spatial diurnal variability of cold stress we can infer that January exhibits more widespread and prolonged cold stress that lasts longer into the morning hours. This confirms that January experiences the most intense, extensive and long-lasting cold stress across north India among all the winter months, while November shows a weaker and shorter cold stress period.

**Fig 4:**
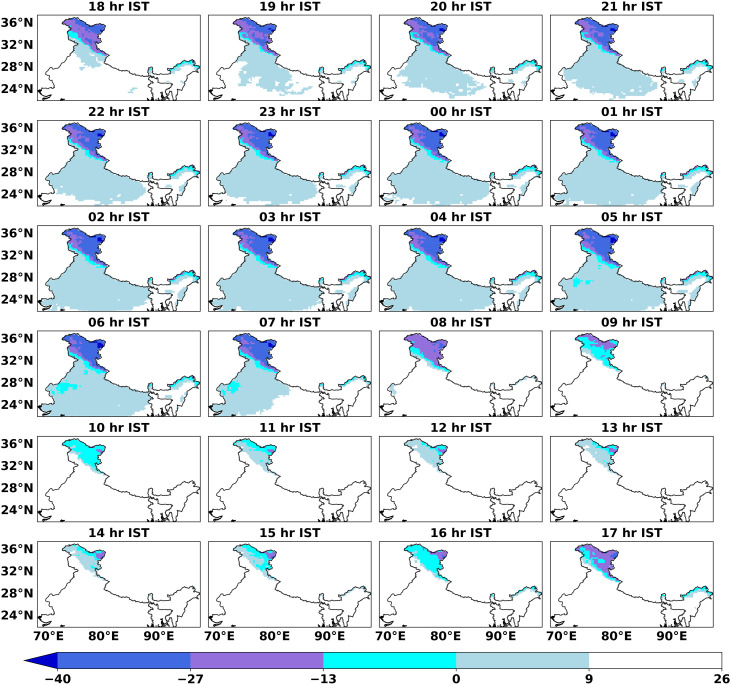
Same as Fig 3 but during cold wave conditions (°C) over the north India. Administrative boundary data were obtained from GeoBoundaries (https://www.geoboundaries.org/) under the Creative Commons Attribution 4.0 International (CC BY 4.0) license. The map was generated by the authors.

Since cold waves bring a significant drop in temperatures, we further analysed the cold stress conditions during cold waves (Fig 4).

During this time, cold stress condition prevails across the entire north India. It initiates around 18 IST and slight cold stress spreads very rapidly across the entire north India compared to the other winter days. Moderate cold stress is also observed in parts of Rajasthan during the early morning hours, which is not usually observed during the normal winter days. During the cold wave, the high altitude areas of J&K and Ladakh are under the grip of strong to extreme cold stress conditions from 18 IST to 8 IST. Kumar et al. [23] observed that in the metropolitan cities of Kolkata and Delhi, people experience slight cold stress during cold waves. This indicates that during a cold wave, both the spatial extent and intensity of cold stress exceed those observed climatologically in January.

### 3.2 Trends in cold stress conditions

**Fig 5:**
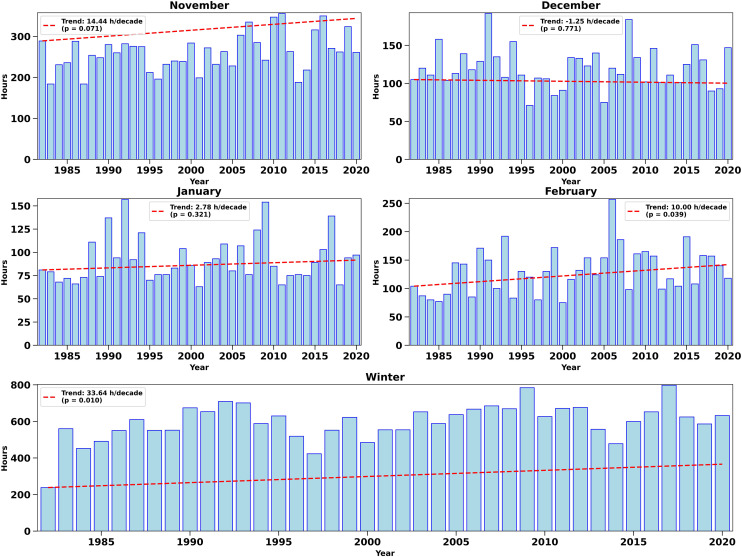
Trend of “slight cold stress (9 °C to 0 °C)” hours from 1982-2020 over north India.

To study the long-term changes in cold stress, we analysed the spatial and temporal trends of different types of cold stress conditions over the selected region for the period 1982–2020. The trend of slight cold (9 °C to 0 °C) stress hours in November from 1982 to 2020 (Fig 5) shows an increase at a rate of 14.44 hours per decade.

In December, the rate of decrease is −1.25 hours per decade, which is weakly significant. In January, slight cold stress exhibits an increasing trend of about 2.78 hours per decade from 1982 to 2020. In February, there is a significant increase at the rate of 10 hours per decade. Overall, during winter from 1982−2020, slight cold stress (9–0 °C) hours are increasing significantly in north India at a rate of 33.64 hours per decade. This indicates even under global warming and a general decrease in the intensity of cold waves [2], cold stress persists in north India, but at a milder level.

**Fig 6:**
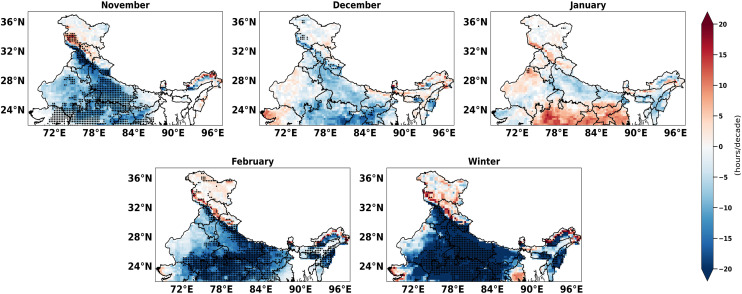
Spatial trend of slight cold stress hours from 1982 to 2020. Administrative boundary data were obtained from GeoBoundaries (https://www.geoboundaries.org/) under the Creative Commons Attribution 4.0 International (CC BY 4.0) license. The map was generated by the authors.

We then analyzed the spatial trend of slight cold stress hours for the period 1982–2020 (Fig 6).

In November, there is a significant decrease in slight cold stress hours over most parts of north India, except for some regions in J&K and HP, where a significant increasing trend is observed. In December, slight cold stress hours show a decreasing trend across all of north India. In January, an increasing trend is observed over most parts of north India, whereas parts of UP, Haryana exhibit a decreasing trend. In February, slight cold stress hours show a significant decreasing trend across most parts of north India, but a significant increasing trend is observed in the high altitude areas of J&K, HP and Uttarakhand. Overall, during winter from 1982–2020, there is a significant increase in slight cold stress hours in the high altitude regions and a significant decrease across the rest of north India. It is interesting to see that a comparable elevation-dependent UTCI signal is evident here. The observed spatial trends in slight cold stress hours in north India are physically consistent with earlier findings on the altitudinal control of thermal stress. A similar relationship between elevation and UTCI was reported in the northern Carpathians [45], where cold stress was shown to intensify with increasing altitude due to lower UTCI values at higher elevations. The study demonstrated that rising altitude leads to an increased frequency of cold stress days resulting from lower ambient temperatures and enhanced atmospheric cooling. These findings suggest that, despite regional climatic differences, altitude exerts a dominant and robust control on thermal stress patterns, consistent with results reported for mountainous regions in other parts of the world.

**Fig 7:**
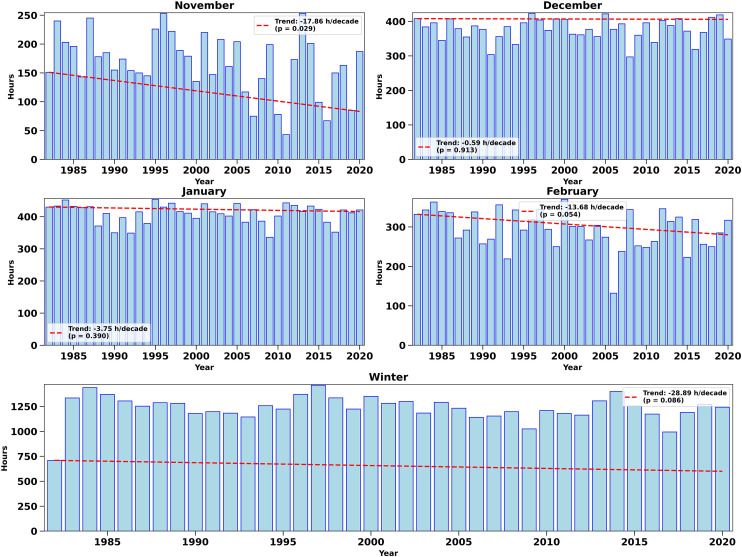
Trend of moderate cold stress (−13°C to 0°C)” hours from 1982 to 2020 over north India.

Similarly, we also examined the trend of moderate cold stress hours during the winter months from 1982 to 2020 over the study region (Fig 7).

Moderate cold stress hours show a significant decrease at the rate of −17.86 hours per decade in November. In December and January, it shows a non-significant decreasing trend at rates of −0.59 and −3.75 hours per decade, respectively. In February, moderate cold stress hours show a significant decreasing trend at a rate of −13.68 hours per decade. Overall, during the winter months, moderate cold stress hours show a non-significant decreasing trend over the study region at a rate of −28.89 hours per decade.

**Fig 8:**
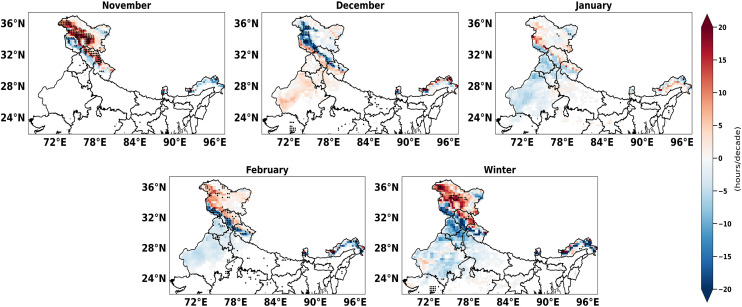
Spatial trend of moderate cold stress hours from 1982 to 2020. Administrative boundary data were obtained from GeoBoundaries (https://www.geoboundaries.org/) under the Creative Commons Attribution 4.0 International (CC BY 4.0) license. The map was generated by the authors.

The spatial trend of moderate cold stress hours from 1982–2020 (Fig 8) shows a significant increase in the high altitude areas of J&K and Ladakh in November.

In December, an increasing trend is observed in parts of northwest India, whereas in January and February, a decreasing trend is noted in the same region. Overall, moderate cold stress hours show a decreasing trend in parts of northwest India and an increasing trend in the J&K and Ladakh region during winter. This spatial pattern suggests a warming trend in the plains and an intensifying cold stress in the mountainous regions. The overall decrease in the moderate cold stress in the plains of north India during the period 1982–2020 can be attributed to the rise in minimum temperatures [2,46,47] and a decrease in the incidence of strong and extreme western disturbances [48]. Similar contrasting behaviour in wintertime UTCI trends has been reported in Japan, where increasing trends were mainly observed in southern and western urban areas, while decreasing trends were concentrated in northern and mountainous regions [49].

Ullah et al. [50] reported an increase in the frequency of slight cold stress days in the Himalayan region, while simultaneously noting a weakening trend in the intensity of slight cold stress. This contradiction reflects a transition from stronger cold stress categories to the slight cold stress category, consistent with our results. Such a shift indicates a reduction in cold severity rather than an intensification of cold conditions. The increase in slight cold stress days can therefore be attributed to a decline in strong and moderate cold stress events driven by a decrease in the frequency, duration, and intensity of cold waves associated with rising winter minimum temperatures [2] and a reduction in the occurrence of very cold days and nights in mountainous regions [51]. As a result, many previously severe cold stress days now fall under the slight cold stress category. To combat the effects of cold stress, residents in north India are encouraged to dress in multiple layers, particularly in the morning and evening. Ensuring access to well-insulated shelters and heating systems in affected regions can significantly lower health risks during winter. Furthermore, limiting outdoor activities during the early morning and late evening is advised [52].

### 3.3. Mortality risks associated with Cold Waves

**Fig 9:**
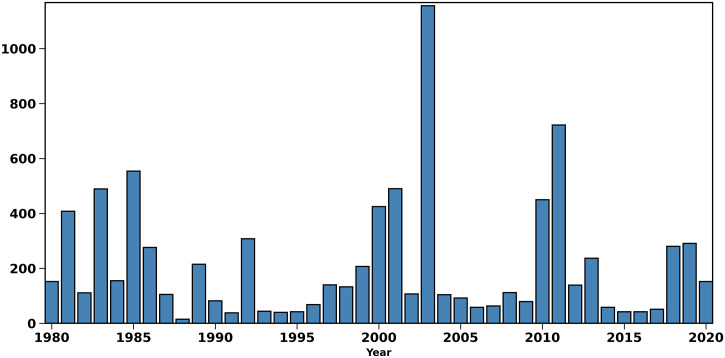
Cold wave mortality in north India during the period 1980−2020.

To study the impact of cold weather and the stress it imposes on human well-being, we examined the mortality risks associated with cold waves in north India. Despite a general decline in the frequency of cold spells globally since 2000, their public health impact remains substantial. At the global scale, most excess cold spell-related deaths are concentrated in Asia, particularly in densely populated regions of southern and eastern Asia, including India [53]. Fig 9 represents the cold wave mortality in north India during the period 1980–2020.

**Fig 10:**
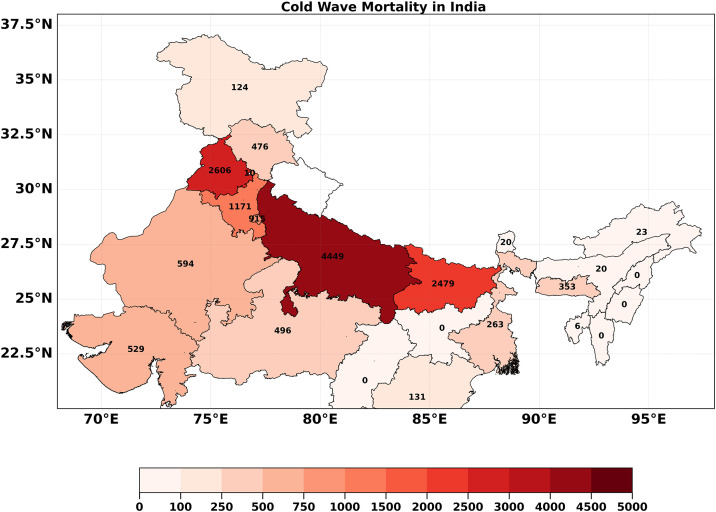
Mortality due to cold waves in north India from 2000 to 2020. Administrative boundary data were obtained from GeoBoundaries (https://www.geoboundaries.org/) under the Creative Commons Attribution 4.0 International (CC BY 4.0) license. The map was generated by the authors.

This signifies that every year, cold waves and the associated stress contribute to a significant number of deaths in northern India. In the year 2003, a cold wave resulted in the death of 1156 people, with the highest number of casualties in UP. Fig 10 shows the spatial variation of mortality across north India from 2000 to 2020, highlighting that the maximum deaths happen in the northern states of UP, Bihar, Punjab, and Haryana.

**Fig 11:**
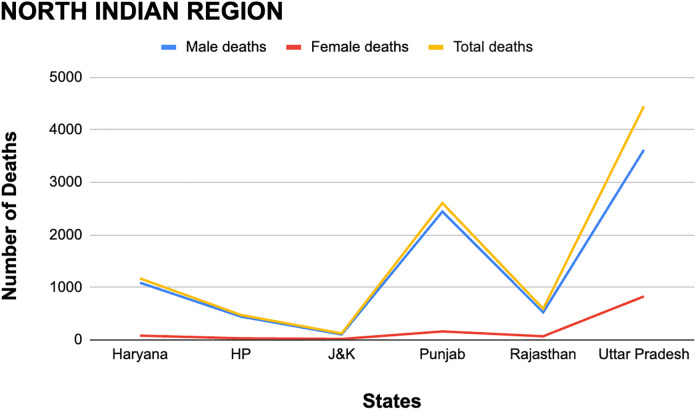
Cold wave mortality in north India from 2000 to 2020. Blue, red and orange lines represent male, female and total cold wave mortality respectively.

From 2000–2020, UP reported the highest death toll of 4449, followed by Punjab (2606 deaths) and Bihar (2479 deaths). Haryana (1171 deaths) and Rajasthan (594 deaths) were also severely affected. Across these states, males exhibited significantly higher mortality rates than females (Fig 11), indicating a clear gender disparity. This is likely due to increased occupational exposure among men, especially those working outdoors as daily wage labourers in these regions.

**Fig 12:**
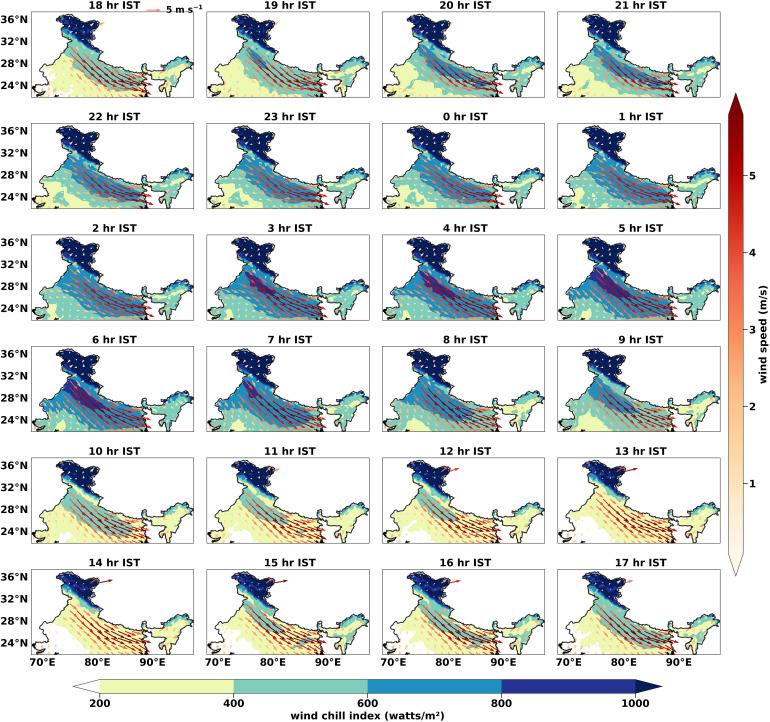
Diurnal variations of the wind chill index during cold stress conditions due to cold waves from 1982 to 2020. Administrative boundary data were obtained from GeoBoundaries (https://www.geoboundaries.org/) under the Creative Commons Attribution 4.0 International (CC BY 4.0) license. The map was generated by the authors.

It is to be noted that the states of UP, Punjab and Haryana have the maximum male mortality and the highest difference in male and female cold wave-related mortality. In contrast, the gender difference in mortality is much smaller (less than 500) in J&K, HP, and Rajasthan. A one-degree Celsius decrease in average winter minimum temperatures within the 5–10 °C range was associated with an additional 13 total deaths (p < 0.05). Males were found to be 21 times more susceptible than females (p < 0.05), and this heightened vulnerability among men was evident across multiple age groups [21]. The states in the Indo-Gangetic Plain (IGP) predominantly experience slight cold stress conditions during winter. Yet, even under such conditions, the mortality rates are high, mainly due to dense populations and poor socio-economic conditions. A study from China reported a significant increase in mortality associated with longer and more intense cold spells, particularly when these events occurred earlier in the season [54]. In addition to these factors, we investigated the specific meteorological phenomena contributing to mortality during cold waves. One such paramount parameter is wind chill, which indicates the combined effect of cold temperatures and wind. Fig 12 shows the diurnal variability of the wind chill index along with the wind speed and direction.

During cold wave events, states in the IGP experience the wind chill effect, which is caused by cold northwesterly winds blowing at speeds of 3–5 m/s. These winds advect cold, dry air into the region, which enhances the chilling sensation experienced by people [29]. In the IGP states, ‘Cold comfort’ with ‘600 < K ≤ 800’ is experienced from 19 IST, which intensifies into ‘Tonic - very cold sub comfort’ from 3 to 7 IST, where ‘800 < K ≤ 1000’. This coincides with the strongest and most widespread northwesterly flow of very cold and dry wind. The persistence of these winds amplifies cold stress due to wind chill. In the densely populated and socio-economically disadvantaged IGP states, this effect significantly contributes to high mortality during cold wave events. Hence, wind chill is a critical factor that exacerbates the impact of cold stress and contributes to increased mortality in the states of UP and Bihar.

The severity of this impact is further compounded by the limited physiological and social preparedness of the population. People born and raised in warmer climates tend to be less physiologically adapted to cold conditions [55]. This may also apply to people in north India, although they are exposed to seasonal cold temperatures. People with lower resilience to cold, such as the elderly residing in areas with fewer adaptation and mitigation strategies [56] for coping with cold weather are at greater risk. This makes welfare measures, such as night shelters and access to alternative energy sources for heating. Night shelters, in particular, have served as crucial interventions to prevent deaths due to extreme cold, especially among vulnerable populations such as poor women, children and the homeless. However, the safety and quality of these shelters remain under concern. In recent years, states like Punjab, Delhi, UP and Bihar have taken steps to increase the number and improve the conditions of night shelters [21].

The impact of cold waves is especially severe among economically disadvantaged communities. Daily wage labourers are particularly vulnerable as their outdoor work is disrupted during cold events [57,58]. The lack of indoor heating further aggravates their situation. In Delhi, large variations in indoor temperatures were observed both within and between dwellings, with the greatest fluctuations occurring in katcha dwellings constructed from temporary materials, reflecting their poor thermal insulation. Across all housing types among low-income households, indoor temperatures exceeded the comfort threshold of 21 °C for less than 40% of winter hours, indicating widespread occupant discomfort and an elevated risk to health from prolonged exposure to cold conditions [59]. Unlike colder mid-latitude countries, most households in north India lack central heating systems or electric room heaters [60]. Dependence on biomass fuels, kerosene heaters, or open fires not only provides insufficient warmth but also increases indoor air pollution, compounding respiratory stress during cold events, leading to respiratory and tract infections [58]. Thus, cold wave mortality in north India cannot be attributed solely to temperature extremes but must be understood as the outcome of compound climatic and socio-economic stressors. Additionally, poor visibility due to the formation of fog during winter impacts daily activities and road safety during the winter months [61]. Since the effects of cold are both delayed and prolonged, the number of people exposed to moderately cold temperatures has increased since 1981 in India [27], highlighting the growing urgency of addressing cold wave impacts in vulnerable regions.

## 4. Summary and conclusion

Minimum temperatures in north India drop significantly during the winter months from November to February. This makes the region highly vulnerable to cold waves and associated cold stress conditions, ranging from slight to extreme levels. Considering their substantial impact on human well-being, the patterns and variability of cold stress are analysed using the UTCI, a biometeorological tool for assessing the thermal stress. During winter, cold stress of various categories is predominant over almost the entire north India. The analysis revealed that cold stress is most pronounced during the late evening (18 IST) to early morning (7 IST) hours, with peak winter month (January) showing the longest and most intense duration of cold stress. During cold wave events, the intensity and spatial extent of cold stress increase significantly, with strong to extreme cold stress noticed in high-altitude regions of J&K and Ladak, and some isolated locations of Rajasthan witnessing moderate cold stress.

Furthermore, the trends of the number of hours of different types of cold stress prevalent in north India have been explored. The trend analysis depicts that slight cold stress hours are increasing significantly across north India especially in high-altitude regions, at a rate of 33.64 hours per decade. Meanwhile, moderate cold stress shows a non-significant but consistent decrease in much of the north Indian region. However, people in mountainous regions (J&K and HP) continue to face cold stress. Given the implications of cold stress, residents are advised to wear multiple layers of clothes, ensure adequate shelter and heating, and limit outdoor activities during peak cold hours.

Lastly, the study also explored the impacts of cold waves on human health, particularly in terms of mortality risks. The highest cold wave-related deaths were reported in UP (4449), followed by Punjab (2606), Bihar (2479), Haryana (1171), and Rajasthan (594). Males experienced significantly higher mortality than females, especially in UP, Punjab and Haryana, likely due to greater occupational exposure and socio-economic vulnerability. Although the IGP primarily experiences only slight cold stress, mortality rates remain high due to dense populations and poor living conditions. Wind chill with a category of ‘Tonic- very cold sub comfort’ caused by cold northwesterly winds further exacerbates the effects of cold stress in these regions.

Although recent years have seen improvements in shelter facilities for people, challenges remain. Vulnerable groups such as the homeless, daily wage labourers and the elderly are impacted, with cold waves also contributing to respiratory infections and increased risk of road accidents due to fog and poor visibility during winters. These findings show the urgent need for comprehensive and targeted cold wave response strategies. Such measures should include the expansion and upgrading of night shelters, provision of reliable heating options, improved clothing and insulation awareness, and public advisories to limit exposure during high-risk cold stress hours.

The Delhi Urban Shelter Improvement Board provides night shelters for the urban homeless during Delhi’s harsh winters. However, despite their availability, many homeless individuals remain unaware of these facilities or avoid them due to distrust, rigid rules, substance dependence, concerns over personal safety and belongings, and discomfort with shared spaces, highlighting persistent systemic and social barriers to shelter use [62]. Strengthening healthcare systems to handle cold-related illnesses, improving access to heating and implementing early warning systems can significantly reduce health risks during winter. Therefore, adaptation programs should focus on the at-risk groups to mitigate the adverse effects of cold waves [8].

## Supporting information

S1 Fig Diurnal variability of cold stress in November (°C). Administrative boundary data were obtained from GeoBoundaries (https://www.geoboundaries.org/) under the Creative Commons Attribution 4.0 International (CC BY 4.0) license. The map was generated by the authors.
(JPG)

S2 Fig Diurnal variability of cold stress in December (°C). Administrative boundary data were obtained from GeoBoundaries (https://www.geoboundaries.org/) under the Creative Commons Attribution 4.0 International (CC BY 4.0) license. The map was generated by the authors.
(JPG)

S3 Fig Diurnal variability of cold stress in February (°C). Administrative boundary data were obtained from GeoBoundaries (https://www.geoboundaries.org/) under the Creative Commons Attribution 4.0 International (CC BY 4.0) license. The map was generated by the authors.
(JPG)
