# Supplementary figures and images for: Cold stress during winter over North India: Patterns, trends, and mortality risks
Source: PLoS One. 2026 Jun 22;21(6):e0351740. doi: 10.1371/journal.pone.0351740 (PMC13286149; doi:10.1371/journal.pone.0351740)

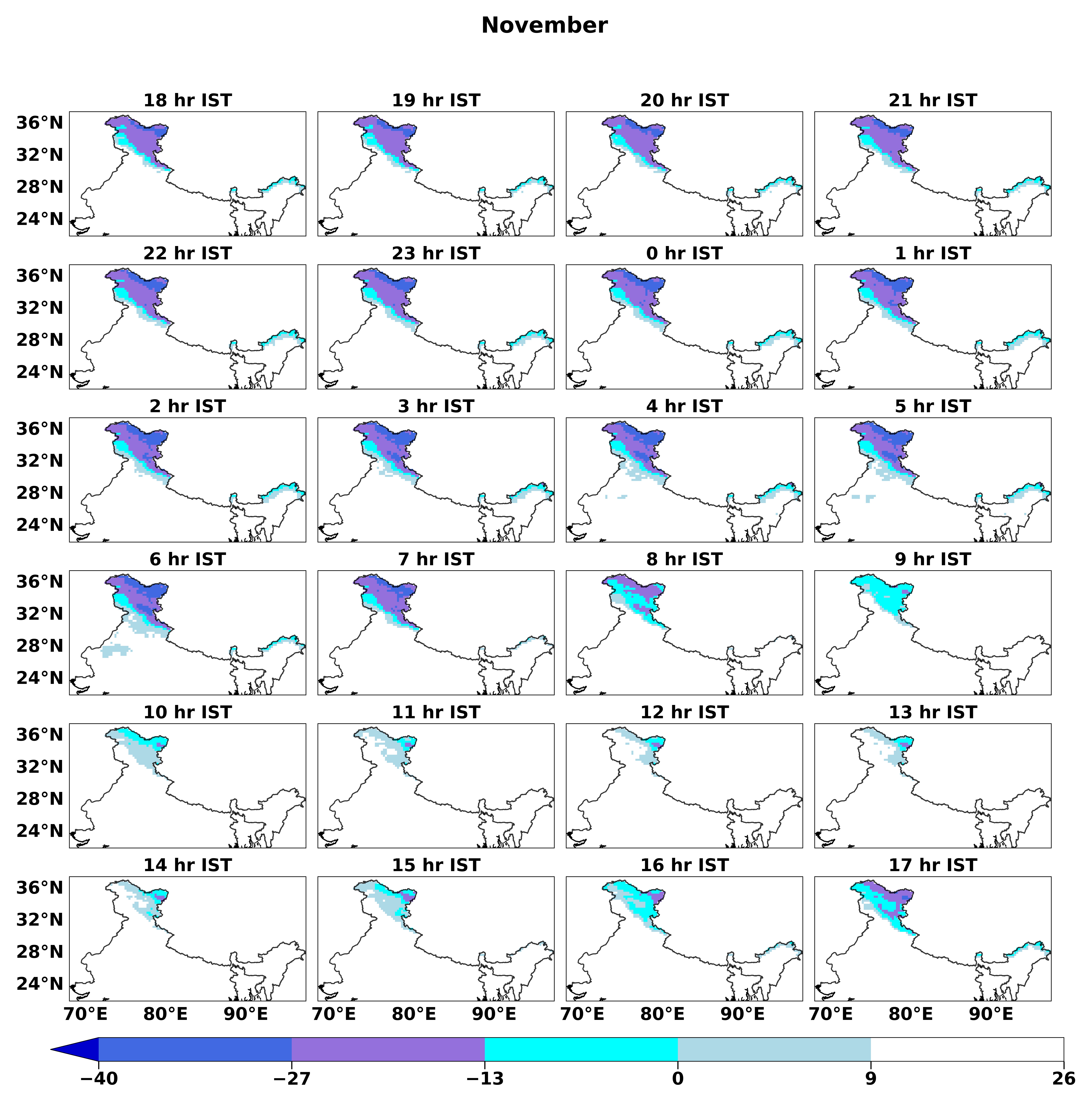

Supplement: S1 Fig — Administrative boundary data were obtained from GeoBoundaries (https://www.geoboundaries.org/) under the Creative Commons Attribution 4.0 International (CC BY 4.0) license. The map was generated by the authors. (JPG) [file pone.0351740.s001.jpg]

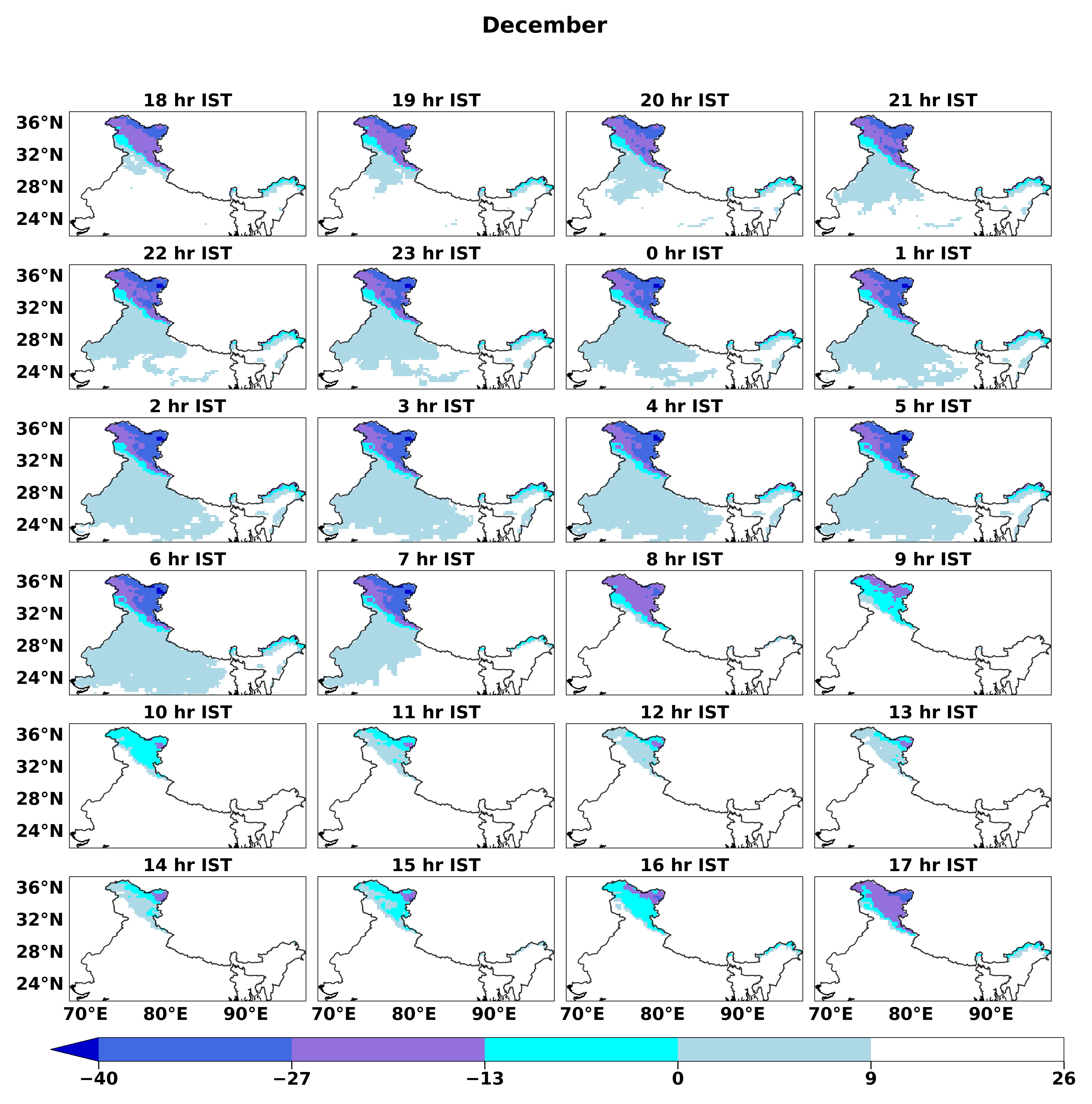

Supplement: S2 Fig — Administrative boundary data were obtained from GeoBoundaries (https://www.geoboundaries.org/) under the Creative Commons Attribution 4.0 International (CC BY 4.0) license. The map was generated by the authors. (JPG) [file pone.0351740.s002.jpg]

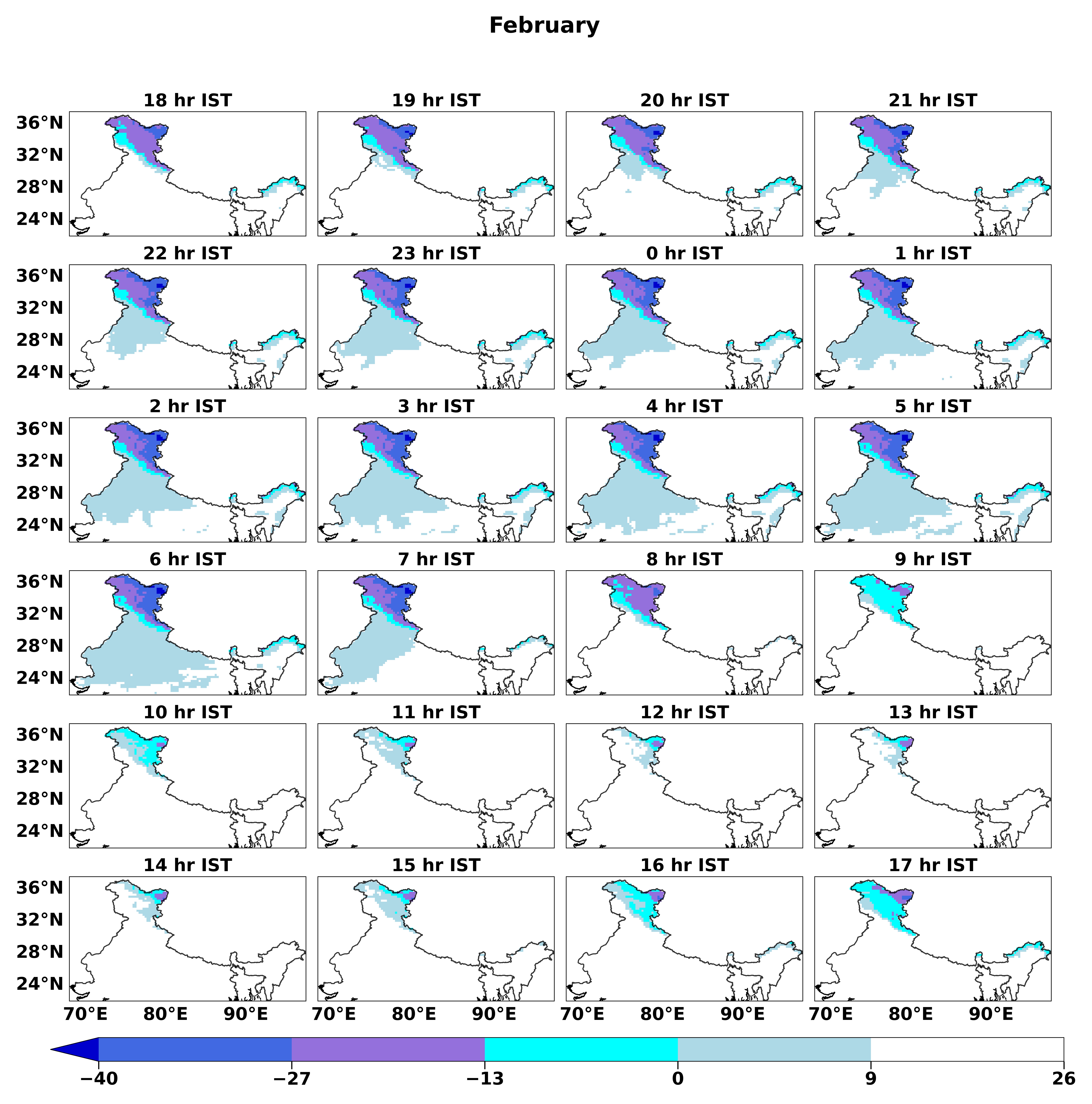

Supplement: S3 Fig — Administrative boundary data were obtained from GeoBoundaries (https://www.geoboundaries.org/) under the Creative Commons Attribution 4.0 International (CC BY 4.0) license. The map was generated by the authors. (JPG) [file pone.0351740.s003.jpg]
